# Integration of Material Characterization, Thermoforming Simulation, and As-Formed Structural Analysis for Thermoplastic Composites

**DOI:** 10.3390/polym14091877

**Published:** 2022-05-04

**Authors:** Philip Bean, Roberto A. Lopez-Anido, Senthil Vel

**Affiliations:** Advanced Structures and Composite Center, University of Maine, Orono, ME 04469, USA; rla@maine.edu (R.A.L.-A.); senthil.vel@maine.edu (S.V.)

**Keywords:** composite, thermoplastic, thermoforming, simulation

## Abstract

An improved simulation-based thermoforming design process based on the integration of material characterization and as-formed structural analysis is proposed. The tendency of thermoplastic composites to wrinkle during forming has made simulation critical to optimized manufacturing, but the material models required are complex and time consuming to create. A suite of experimental methods has been developed for measurement of several required properties of the molten thermoplastic composite. These methods have the potential to enhance thermoplastic composites manufacturing by simplifying and expediting the process. These material properties have been verified by application to thermomechanical forming predictions using commercial simulation software. The forming predictions showed improved agreement with experimental results compared to those using representative material properties. A tool for using thermoforming simulations to inform more accurate structural models has been tested on a simple case study, and produced results that clearly differ from those of models using idealized fiber orientations and thicknesses. This provides evidence that this type of as-formed analysis may be necessary in some cases, and may be further investigated as an open source alternative to commercial analysis software.

## 1. Introduction

**Figure 1:**
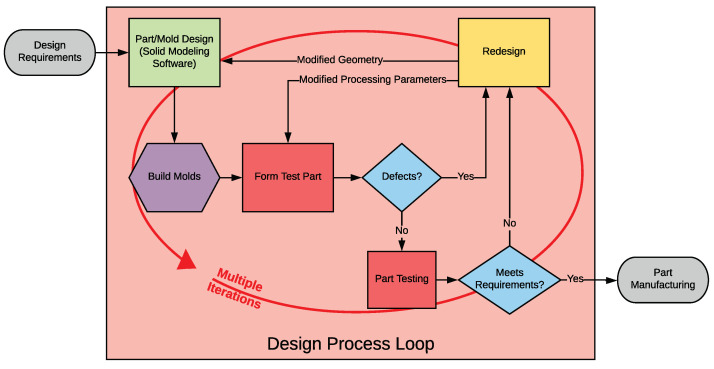
Flowchart of the traditional thermoforming design process.

**Figure 2:**
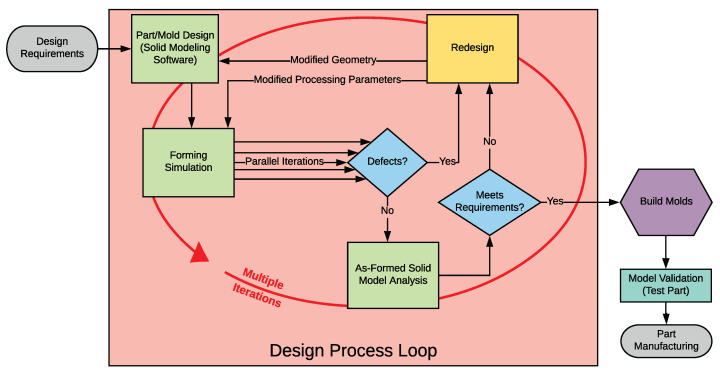
Flowchart of the simulation-based thermoforming design process.

Continuous fiber reinforced thermoplastic composite materials (CFRTPs) contain fiber reinforcement in a meltable thermoplastic matrix, which differs from the more common thermoset composites in which the reinforcing fibers are embedded in a matrix of a chemically-cured resin. This difference gives CFRTPs some unique advantages including higher fracture toughness than their thermoset counterparts [1]; better recyclability [2]; a variety of rapid-manufacturing technologies; and the ability to assemble or repair by welding [3] (in addition to conventional methods like adhesive-bonding or bolting). Thermoplastics also have the ability to be manufactured by a process called thermoforming, wherein a flat thermoplastic composite ‘blank’ is heated and pressed to shape between press-molds. This versatile process allows for rapid production with cycle times as low as five minutes [4,5], which is much faster than typical thermoset processes. One of the largest drawbacks to thermoforming is the difficulty in forming complex shapes without defects, due to the composites being nearly inextensible along the fiber directions [6,7,8,9]. This forces the material to conform to a shape almost exclusively by shear deformation, which has a tendency to cause wrinkles that can compromise the structural integrity of the part. These kinds of defects can often be mitigated by altering the forming process with different temperatures, speeds, or simpler geometry [10,11,12]. These alterations have traditionally been implemented in a “trial and error”-type manner as shown in Figure 1. This can be costly and time consuming due to the need for iterative physical manufacturing [13,14,15]. In addition, it requires a high level of experience-based intuition. In order to increase process efficiency, then, the trial and error method has been replaced by computer simulations. In fact, the Consortium for Manufacturing Innovation in Structural Thermoplastics (CMIST) concluded during roadmapping workshops that simulation software and experimental characterization of the requisite material processing properties are a significant need [16]. To be useful, these simulations must accurately predict a variety of behaviors including wrinkling and fiber reorientation. These predictions can allow the costly manufacturing processes to be removed from the design loop (as shown in Figure 2), thus improving efficiency, and allowing for better process optimization. This simulation-based design process has recently been gaining traction in industry as the simulation tools have matured [14].

The goal of this research is to aid in the development of simulation methods, which can help this promising technology become more widespread. This work then approaches that goal in a threefold manner. First, to collect a novel set of methods for characterizing material properties for forming analysis. Second, to verify the usefulness of these material properties in thermomechanical forming predictions using commercial simulation software. Third, to extend the use of simulation results for use in structural analysis of the as-formed part.

In order to properly simulate the forming process, material properties must be measured accurately at the processing temperature. These properties can be measured using a standard tensile test frame in a variety of ways including tests like the picture-frame shear test [6,7,17,18,19,20,21,22] or bias extension test [20,23,24,25,26]. Unfortunately, this can present some difficulty, as it requires the use of a high temperature environment chamber so the experiments can be performed at the material forming temperature. Therefore, a set of experimental methods has been proposed to simplify the measurement of these properties. These experiments utilize dynamic mechanical analysis (DMA) techniques.

These material characterizations needed verification to be used in simulations. To this end, two case-study parts were simulated using industrial simulation software, and the results were compared against actual formed parts. A commercial simulation software package was chosen in order to focus on the integration of the entire process, rather than on developing new software. An additional benefit of this is that the methods discussed here could be easily implemented into an existing workflow.

Forming simulation results, while traditionally only used for tuning the manufacturing process, also contain detailed data regarding the final as-formed part. Rather than ignore this data, it is also useful to inform a structural analysis of the as-formed part, since fiber alignment changes during forming [27,28,29,30]. To this end, we developed a tool that uses the local fiber alignment and thickness predictions from the forming simulation to build a structural model. This is expected to better predict the final part’s performance than a structural model that uses the initial ply thicknesses and fiber orientations, since fiber angle and volume fraction are significant factors in composite material behavior. Thus, the usefulness of a forming simulation is extended.

## 2. Experimental Characterization Methods

In order to accurately simulate forming, several material properties must be known. These are primarily in-plane properties of the laminae (layers). Here, the focus is on the in-plane shear modulus ($G_{12}$), the tensile moduli in longitudinal (fiber) and transverse directions ($E_{1}^{t}$ and $E_{2}^{t}$), and the bending moduli in the longitudinal and transverse directions ($E_{1}^{b}$ and $E_{2}^{b}$). While there have been many different methods proposed for measuring these properties, we chose to use a set of dynamic mechanical analysis (DMA) experiments. DMA, despite being well established as a method, and having several advantages over alternative methods, has not been utilized extensively in the thermoforming field. The primary benefits of DMA are as follows: First, DMA machines typically have high temperature thermal control thus eliminating the need to fit a tensile test frame with an environment chamber. Second, the oscillatory nature of the DMA process allows for a single specimen to be tested continually through a wide range of temperatures and strain-rates, thus decreasing the number of experiments needed. Third, DMA samples are much smaller than typical specimens, thus further decreasing the amount of material needed for characterization. The data from these dynamic experiments comes in the form of the ‘storage modulus’ and ‘loss modulus’. The storage modulus is of interest to this work as it can serve as a proxy for the corresponding elastic modulus (tensile, shear, or bending). While not investigated here, the loss modulus is useful in characterizing the viscous behavior of the material. The polymer material selected was polypropylene, which is a commodity semi-crystalline thermoplastic. These experiments were performed using unidirectional E-glass fiber reinforced polypropylene (Avient prepreg unidirectional tape IE 6034) for use with an automated tape layup machine (hereafter referred to as “PP/glass”). These tapes have a nominal fiber content of 65% by weight. The various experiments are summarized in Table 1 and detailed below.

### 2.1. Shear Modulus

Due to the high longitudinal modulus, continuous-fiber reinforced composites must rely on their ability to deform in an in-plane shear mode (shear strain $\gamma_{12}$) in order to conform to complex geometries [6,21,24]. This phenomenon can be avoided by using chopped-strand, knitted, or stretch-broken (aligned discontinuous) fibers. Each of these is more formable than (equivalent) continuous fiber materials because of their ability to stretch in-plane. These are generally not used in high performance applications due to their decreased mechanical properties; however, some stretch-broken-fiber materials have been shown to have strength values comparable to equivalent continuous-fiber systems [31,32,33]. The importance of shear modulus has prompted investigation of a variety of measurement methods, including picture-frame, bias-extension, and torsion experiments. It has been shown to be difficult to adapt the former two methods to unidirectional materials [34,35,36]; however, Haanappel et al. [35] have found promising results using torsion experiments. For this reason, torsional rheometry was used in this research.

**Figure 3:**
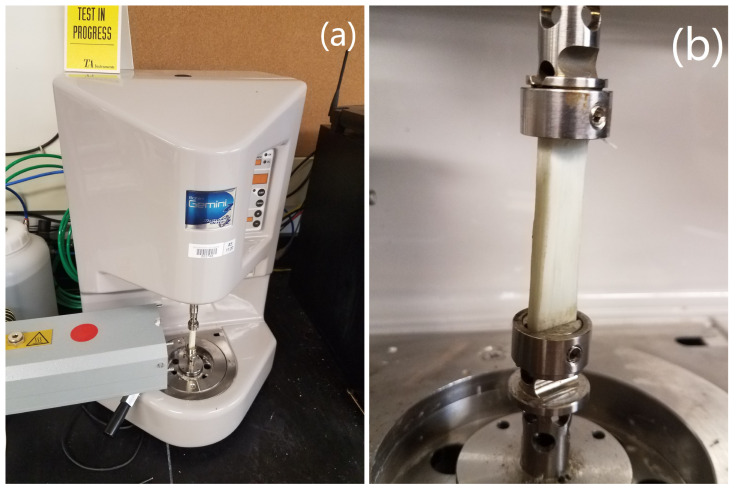
Photos of the torsional rheometer experimental setup. (**a**) Bohlin rheometer with ETC; (**b**) 10 mm × 4 mm × 35 mm specimen installed in fixture clamps.

Torsion experiments were performed on a Bohlin Gemini II rheometer (Figure 3) using the extended-temperature cell (ETC) in order to achieve the desired conditions. This machine was used to run an oscillatory strain-controlled deformation while sweeping through different temperatures or frequencies. These tests were first performed using the temperature sweep, and then later using frequency sweeps at the various temperatures in order to fully capture the behavior and inform a data lookup table for the simulation. The samples measured were unidirectional PP/glass composite manufactured by stamp-thermoforming to consolidate 18 layers into a 4 mm thick panel. This thermoforming process involved pressing a 180–190 ${}^{{}^{\circ}}$C blank between ambient-temperature platens at a target of 0.69 MPa (100 psi) surface pressure for 1–5 min until cool. The resulting panels were then cut into samples using a CNC waterjet cutter. After cutting, the samples were then conditioned in a standard laboratory environment (23 ${}^{{}^{\circ}}$C and 50% humidity) for a minimum of 40 h in accordance with ASTM D618-13 Procedure A [37]. Two sample geometries were used: a 10 mm by 4 mm rectangular prismatic section, and a dogbone with a 4 mm square prismatic gage section. Both samples had a 35 mm gage length.

**Figure 4:**
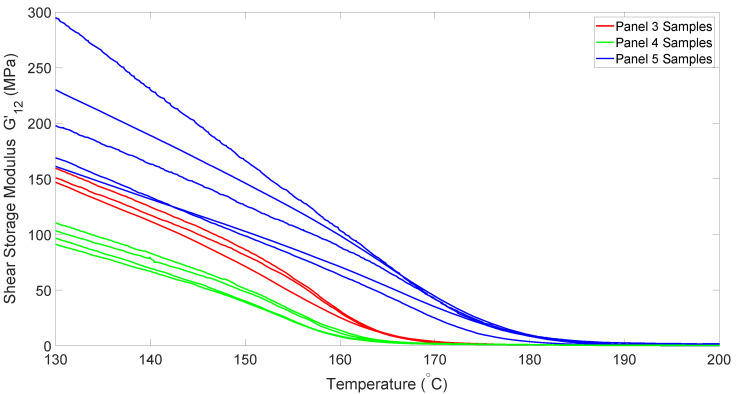
Shear modulus for samples from different panels as a function of temperature.

When analyzing the heating-curve data gathered using samples from different panels, it is clear that there are significant differences between each panel. In Figure 4, we can see that each dataset from any given panel varies only slightly from the others, whereas the differences between panels is quite large. It is speculated that this variation may be driven by crystallinity differences due to cooling-rate changes when the panels were consolidated. Since these panels were manufactured as separate batches, variations in thermal history are likely. For the purposes of this research, we decided to simply average the data from each individual panel, and use them as separate datasets.

### 2.2. Tensile Modulus

The longitudinal tensile modulus in unidirectional thermoplastic composites tends to be orders of magnitude higher than the shear modulus at processing temperatures, thus mandating that the material will deform primarily in shear. This means that the accuracy of the longitudinal modulus is much less critical to the model predictions than the shear modulus. The transverse modulus, however, will still be relatively low, therefore accurate measurements are required.

In order to measure these properties, a DMA (Q-800 DMA by TA Instruments) machine was used. This machine, like the rheometer, utilizes an oscillating deformation, and gathers a continuous stream of data on the material response as temperature and other parameters are varied. The only difference being that the DMA machine fixture adopted applies a tensile load rather than a torsional one. For the tension DMA, thin samples were manufactured by hot-pressing single tapes of the same PP/glass in order to remove the spool-curl from the material and to smooth the surface for more reliable thickness measurements. This is similar to the process used for the torsion specimens, with the notable difference that only a single layer was utilized rather than multiple. The resulting panels were approximately 0.3 mm thick. Samples were made to the largest allowable size (8 mm × 30 mm) for measurements in both the longitudinal and transverse directions.

**Figure 5:**
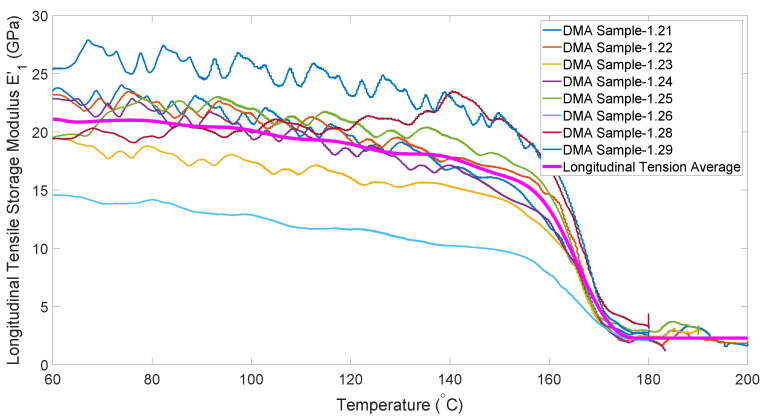
Temperature dependence of longitudinal tensile modulus.

Figure 5 shows the temperature dependence of the longitudinal tensile modulus obtained from the DMA experiments. Note that these values are several orders of magnitude higher than the shear moduli, thus demonstrating the relative inextensibility of the fibers, which forces shear deformation.

**Figure 6:**
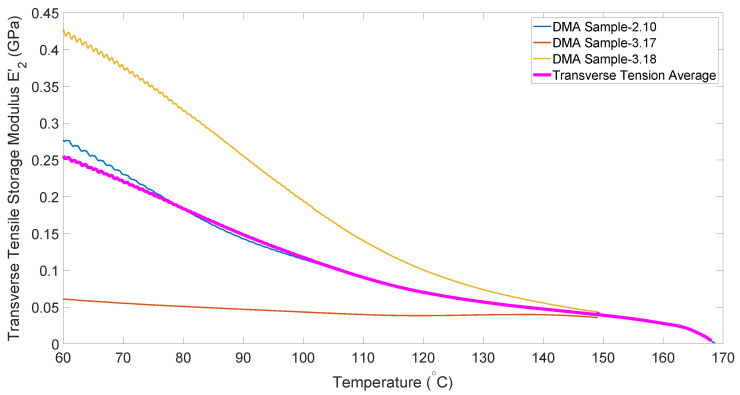
Temperature dependence of transverse tensile modulus.

Figure 6 shows the temperature dependence of the transverse tensile modulus obtained from the DMA experiments. One important difference from the longitudinal data is that the transverse tensile modulus actually goes to zero near the processing temperature (180–190 ${}^{{}^{\circ}}$C), unlike the longitudinal modulus where the fibers provide residual stiffness even after the matrix is molten. It is also worth noting that there is significant scatter in the data. This may be driven by defects in the matrix that also cause some specimens to be quite fragile in this direction.

### 2.3. Bending Modulus

**Figure 7:**
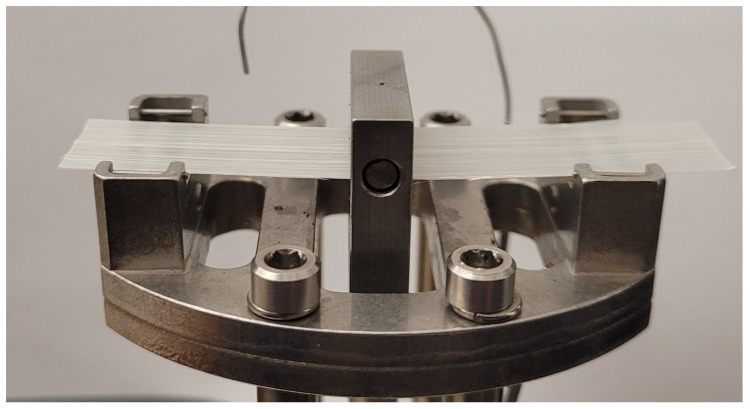
15 mm × 0.3 mm × 50 mm specimen installed in 3-point bending DMA fixture.

The modulus of the individual layers in bending is also an important property, since, while the shear deformation determines the critical areas for wrinkling, the bending is what drives the size and distribution of wrinkles in the high shear areas [11,38,39]. Unlike homogeneous materials, however, the bending rigidity of molten composite laminae cannot be directly inferred from the tensile modulus. Due to interactions between the fibers and matrix as well as fiber migration, the modulus in bending can often be significantly smaller than the corresponding tensile moduli. This necessitates a separate series of experiments for the bending modulus of the tapes. For this, the Q-800 DMA machine was utilized using a 3-point bending fixture (see Figure 7) with 15 mm × 0.3 mm × 50 mm samples, which were manufactured identically to the tension samples. Note also that this unidirectional material had insufficient strength in the transverse direction for any bending experiments. Since the composite in question is unidirectional, there is no reinforcement in the transverse direction, making this tensile-modulus matrix-controlled [40]. Therefore, transverse-bending modulus would also be matrix driven. Since the difference between bending and tensile moduli is a reinforcement-controlled phenomenon, the transverse moduli (tensile and bending) should not exhibit this difference. For this reason, we make the assumption that transverse-tensile modulus can be substituted for transverse-bending modulus.

**Figure 8:**
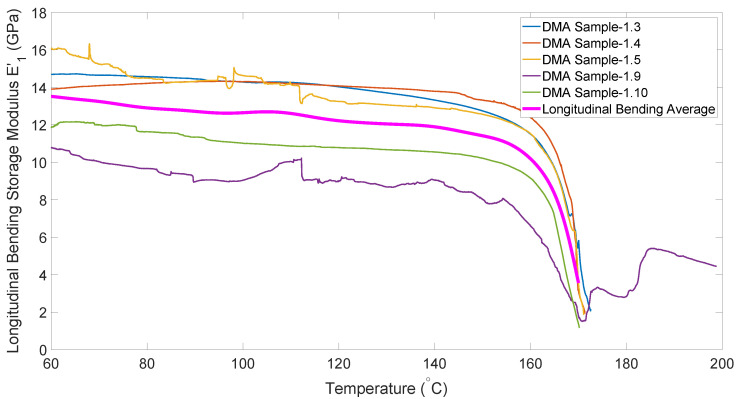
Temperature dependence of the longitudinal bending modulus.

**Figure 9:**
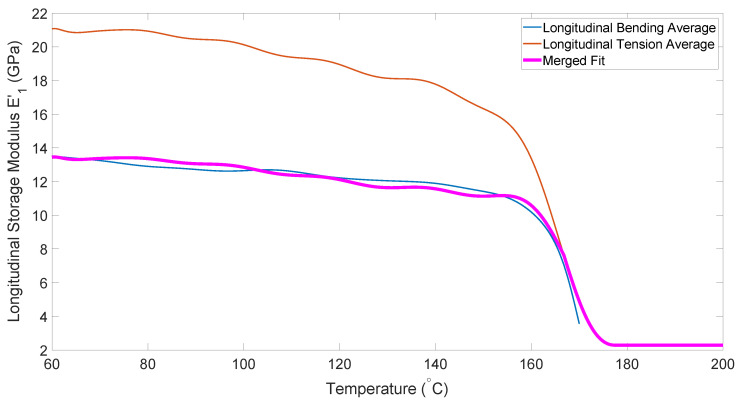
Curve fit for longitudinal bending modulus.

The bending data in Figure 8 follows a similar trend to all previous data, with the exception of its erratic behavior above melt temperature (approximately 170 ${}^{{}^{\circ}}$C). This is due to the sample with molten matrix being too compliant to be stable in the fixture. In order to generate data for use in simulations at processing temperature (180–190 ${}^{{}^{\circ}}$C), the relationship between this curve and the tension curve was used. Figure 9 shows that for much of the heating curve, the bending modulus is lower than the tensile modulus, but near the melt temperature the two curves converge. This behavior was utilized in order to predict the bending modulus at higher temperatures by fitting a curve to follow the bending modulus for lower temperatures, and then smoothly transition to following the tensile modulus.

Some other properties to define include contact interactions and thermal properties. The contact interactions determine how the plies will slide relative to the mold, and relative to each other. Contact in forming is a very complex topic due to the interaction between sliding friction, external pressure, and viscous flow, which all vary with temperature [41,42,43]. Due to the complexity and lack of existing data, we decided to use an estimated constant friction parameter to approximate the contact forming interactions. This was implemented as a constant gluing stress of 1 MPa (which simulates adhesion by forcing plies together once they contact one another), and friction coefficient μ=0.8, which were chosen by qualitative comparison of simulations using various options. Thermal properties are necessary to simulate processes in which thermal gradients and cooling effects are significant (such as rapid processes with fast cooling times). In these instances, the thermal behavior must be accounted and additional properties are needed. These include thermal conductivities of the blank and mold, conductivities of the blank-mold and ply–ply interfaces, and specific heats of the blank and mold. For a large number of forming applications, however, these effects are relatively small and the simulation remains accurate despite idealizing the process as isothermal [44]. In this research, the modeling has been idealized as isothermal for simplicity.

## 3. Thermoforming Simulations

The forming behavior of thermoplastic composites is governed by the complex interaction of the fiber-structure and the matrix viscosity. To completely model this complex system using finite element analysis (FEA), everything would need to be modeled at the meso-scale (scale of fiber-structure). This model would be prohibitively complex for any but the simplest designs, requiring far too much computing time. Commercial forming simulation software bypass this issue by approximating the behavior with the effective properties of the overall composite (i.e., “homogenized” or “average” properties), thus allowing for the use of much larger elements, and producing viable models. Some alternative methods (including kinematic, hybrid, and membrane models ) have also been developed in order to simplify the simulation process [34,39,45,46,47].

In a typical orthotropic material model, Poisson’s effects cause coupling between the longitudinal and transverse stress and strain components in the plane of the lamina. In this work, however, we model the composite as an orthotropic material without any coupling effects. This approximation retains simplicity for setup and computation, while also providing accurate results as seen in [48,49]. The stress–strain relationship in each direction, then, is governed by only the modulus in that direction, and is completely independent of the stress in the other directions. The moduli may also be functions of other variables like strain, strain-rate, and temperature. These variations are implemented in a lookup table, wherein the values of the moduli for each element are chosen based on its current state.

One implementation of this simplified model exists in the industrial software PAM-Form [50], which is an explicit finite element analysis software with additional tools to facilitate forming simulations (such as advanced contact modeling and a dedicated composites material model MAT140). By utilizing this software, we focus on integrating the simulation with the material characterization and later structural analysis, rather than on developing a new simulation tool.

The decoupled modeling is very simple for elastic materials, but additional complexity is introduced by the inclusion of viscoelasticity. While other options exist in PAM-Form, we chose to utilize the “lookup-table” functionality, wherein the modulus is determined by interpolation between stress/strain curves measured at different strain-rates and temperatures; thus capturing a portion of the viscous behavior. This empirical method was chosen due to the directness of the approach, as experimental data can be input directly as a table of values [50].

Advanced contact modeling tools make it possible to simulate forming of multi-layered laminates with inter-ply slip. This capability is one reason that explicit solvers are sometimes preferred over implicit solvers [13,48,51].

### 3.1. Simulation Framework

All models were simulated using 4-noded quadrilateral shell elements. Each individual layer was modeled separately so as to allow inter-ply slip and fiber realignment, though this was moderated by the contact definition, which imposed a friction and gluing stress between the layers and with the tooling. The details of the friction parameters were discussed in Section 2. These models were run isothermally at 180 ${}^{{}^{\circ}}$C using parameters measured in Section 2 and the density, which is derived from published constituent densities and fiber content. The data is summarized in Table 2.

One difficulty in using a software like PAM-Form is that the models can occasionally become unstable. While this can occur in an unpredictable way, it is often caused when significant wrinkles or excessive stresses arise. These can cause the model to crash, as it cannot account for the extremely large stresses and rotations involved, and so stops calculation. These failed models, then, are quite informative. Since the solver is capable of predicting real wrinkles, it follows that wrinkles large enough to crash the solver correspond to significant defects in a real part.

### 3.2. Simulation Case Studies

Two different forming simulations have been performed in this research, both to demonstrate the capabilities of the simulations, and as validation tools to check the correctness of the material characterizations.

**Figure 10:**
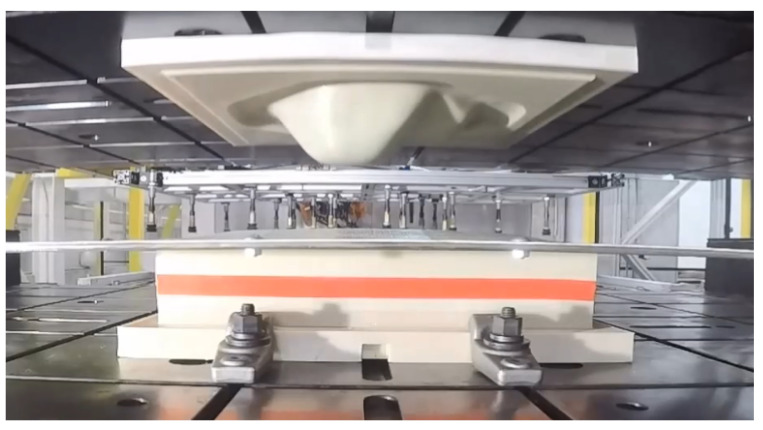
Differential cover mold mounted in press.

**Figure 11:**
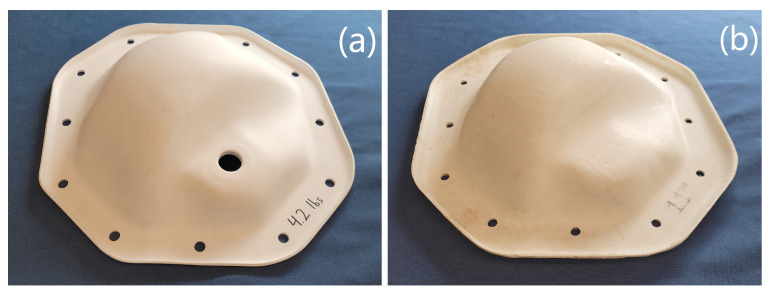
Comparison of 300 mm wide (12″) differential covers. (**a**) Original steel part; and (**b**) thermoformed composite part.

The first case-study is an automotive part. Specifically it is the cover for the rear differential of a pickup truck. The geometry used was derived from measurements of an existing metal part, and direct composite replacements were thermoformed using 3D printed tooling (Figure 10). The original steel part and thermoformed composite part are seen in Figure 11.

**Figure 12:**
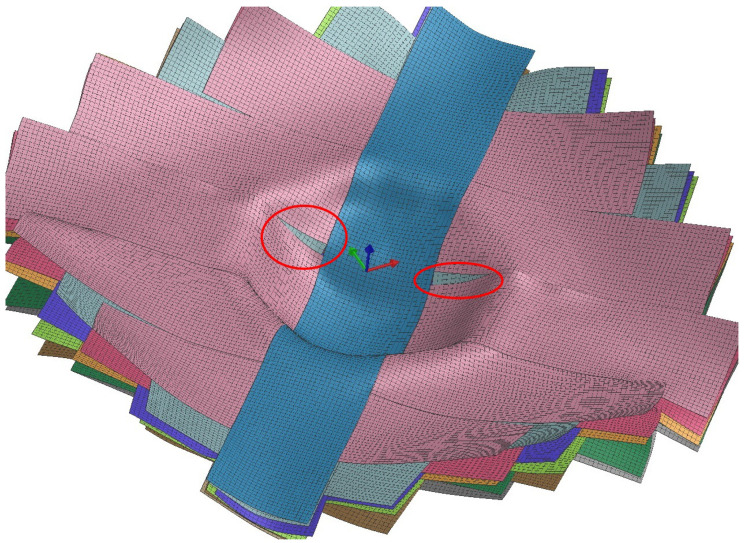
Tape separation exhibited by the differential cover model.

The forming of this part was simulated with 17 layers using a 10 mm target element size. Initial simulations were run using a set of generic-composite properties modified from an example file from PAM-Form. These were later replaced with those measured experimentally as discussed in Section 3.1. Before analyzing the numerical results, qualitative inspection of results reveals some useful information. First, several of the defects exhibited by the real differential cover were also exhibited by the model. For example, the separation between tapes within a layer that was visible in early iterations of the process was also visible in the model (see Figure 12). The figure shows a PAM-Form model result in which the different plies are rendered in different colors. The top layer (blue) is a partial ply that was added to help mitigate the ply separation. Next, we see the first full layer (pink), which has developed gaps that reveal the underlying layer (gray).

**Figure 13:**
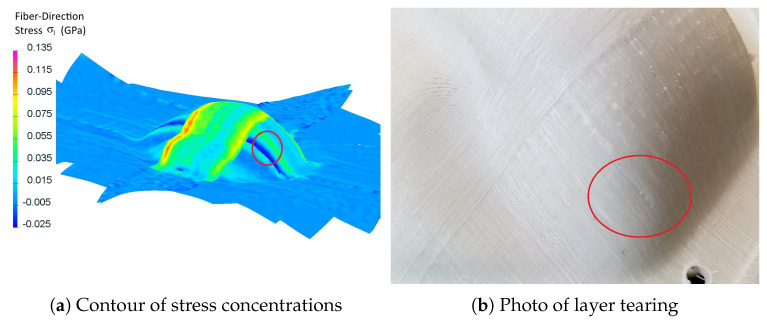
Comparison between simulated stress and forming defects in a differential cover.

Second, stress concentrations appear in an area found to be one critical for ply splitting/tearing (Figure 13). It is unclear what mechanism causes the longitudinal stress banding to translate to transverse splitting, however, the simulation clearly shows that these regions are important in terms of stress concentration. Further investigation of thermoforming failure modes may be of value in furthering the predictive value of the simulation.

Lastly, the general trend of thickness variation at critical pinching locations is exhibited in both model and formed-part.

These qualitative observations appear even in models run using the generic material properties, thus indicating that some useful approximations, and critical-locations can be predicted without full material characterization, and also that certain behaviors are heavily geometry dependent, rather than material dependent. For smaller defects, such as wrinkles, it is assumed that accurate material data is necessary.

In order to validate the models, a numerical comparison was needed for relating the simulated results with experimental ones. The comparison chosen was to use full-field non-contact digital image correlation (DIC) to measure the displacement field in an experimental part (full-size) from before and after forming. This is then converted to a strain field from which shear angle is taken for comparison to simulations [52]. Note that shear angle is simply the shear strain (typically presented in radians) converted to degrees for better readability.

**Figure 14:**
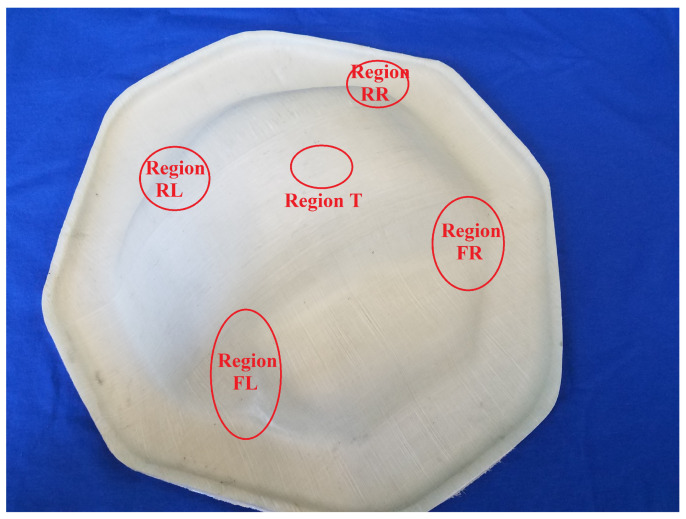
Critical regions in differential cover forming.

The locations chosen for inspection were known critical locations for wrinkling, also being the regions in which the highest shear-angles are observed. In addition to this, the shear angle in the patch (along the crest) was also inspected. See Figure 14 for the locations of these inspection-points.

Using a DIC system, the peak shear-angle magnitude from each of the critical regions was taken. Measurements were averaged from two formed parts. The number of specimens was limited by the amount of available material for forming. These measured values were then compared with those taken from the surface layers of PAM-Form models, thus determining which material model best simulates the actual material. As discussed in Section 2, the data necessitated there be 3 different material models in which the shear modulus ($G_{12}$) is defined using the experimental results of samples from the three different panels (labeled 3, 4, and 5). Table 3 shows the results of this comparison. Note that panel-4 material is omitted due to numerical instability in the corresponding model, which prevented its completion.

From the table, the differences between the different measured properties’ model and the generic properties’ model can be seen.

The first thing to note is that all the models overestimate the shear in the top point of the part. This can be explained in that the peak of the part contacts the mold first, thus facilitating a more rapid cooldown and limiting the shear deformation at this point. Since the simulations treated the process as isothermal, this phenomenon was not captured. For this reason, this point will not be included in the average error measure.

Additionally, with the exception of the top point, the measured material models tend to underestimate the shear, where the generic-material overestimates it. This would suggest that the actual shear modulus in the part is below that found in the measured models, but above that in the generic-material model.

Finally, an average was taken over the magnitudes of the errors from each material model, in order to have a single quantity on which to judge each model’s merit. The results are as follows:

- Generic Material had an average error of 60%;
- Panel-5 Material had an average error of 51%;
- Panel-3 Material had an average error of 43%.

From this, it is clear that the panel-3 material is the best of the models here tested. In addition, since there is an assortment of under- and overpredictions of angle, the error average (rather than average magnitude) shows that holistically, the predictions are slightly better (58%, 48%, and 30%, respectively).

The next case study is a hemisphere, which is a relatively simple shape that also exhibits double-curvature ( where the part curves in multiple directions) as most real-world parts do. Simplicity combined with double-curvature has made the hemisphere a common forming case-study [41]. Our hemisphere was 76.2 mm (3″) diameter, a size which balanced material usage and deformation visibility. By trial and error, the layup and processing parameters were optimized to achieve good consolidation and shape. For this study, a simplified manual process was utilized whereby a preconsolidated blank with a layup of ${\left[\pm 45^{{}^{\circ}}\right]}_{5}$ was placed in a convection oven set at 200 ${}^{{}^{\circ}}$C for 5 min. The softened blank was then carefully transferred to a 10-ton pneumatic press, and stamped between a set of hemispherical aluminum molds. The forming of this part was simulated with 10 layers using a 5 mm target element size.

**Figure 15:**
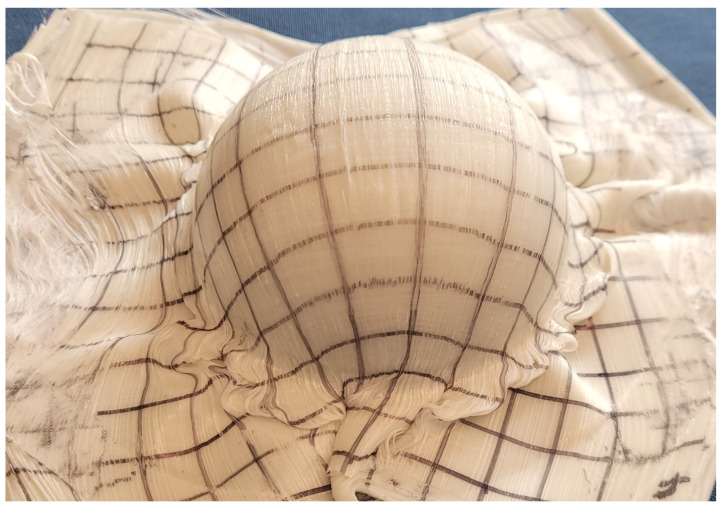
Formed hemisphere.

Due to the mold shape, the material is not restrained at the region of transition between the hemispherical and flat portions of the mold. As a result, the material in the transition region is quite wrinkled (Figure 15). This issue means that the comparisons from model to forming will be restricted to the hemisphere itself, and the adjacent regions will be ignored.

**Figure 16:**
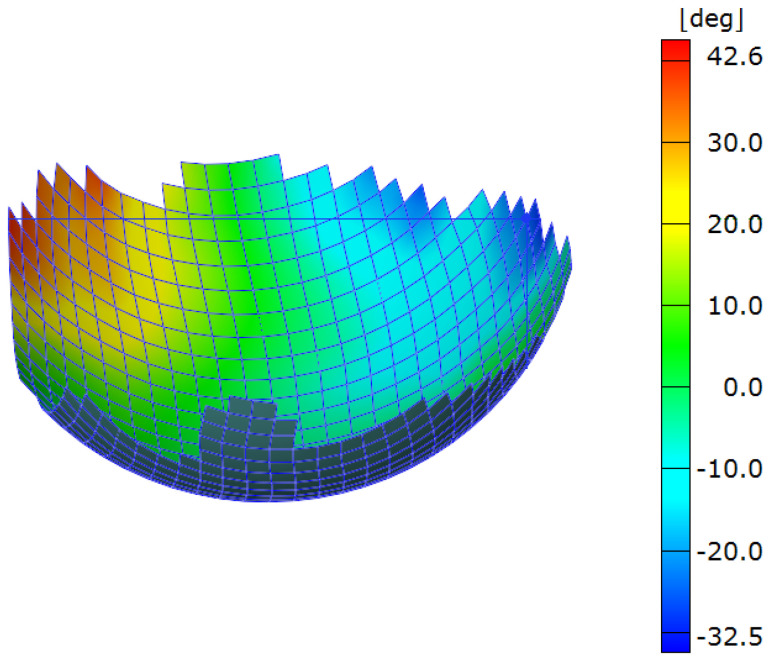
Shear angle field in a hemispherical part.

**Figure 17:**
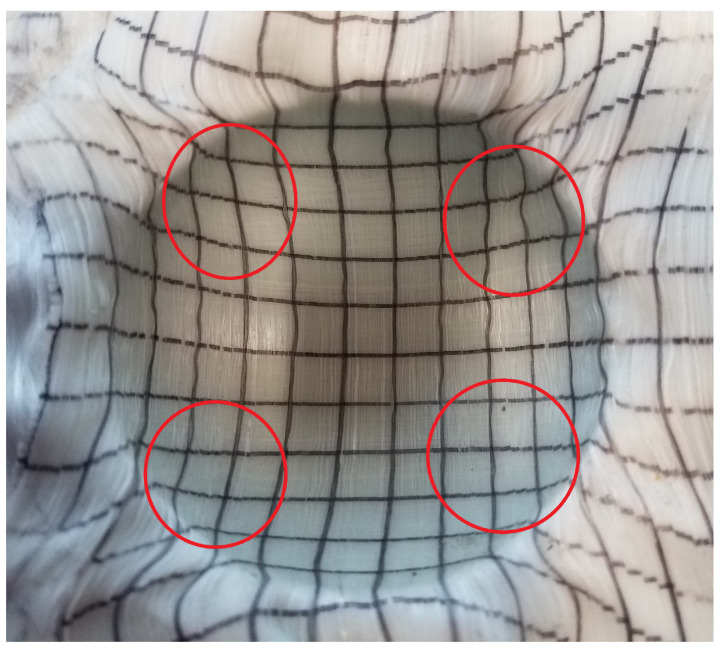
Critical regions in hemisphere forming.

As with the differential cover, hemispherical parts were formed from PP/glass, and shear measurements taken using DIC (Figure 16). These measurements were then compared to simulated results using the several material models (with the exception of M5, which failed by unstable wrinkling behavior). Unlike the differential cover, however, the symmetric nature of this part means that there are no distinct locations to compare. Instead, the peak magnitude of shears in the bias directions (the $\pm 45^{{}^{\circ}}$ directions as shown in Figure 17) were averaged to generate a comparison criterion. The results of the comparison can be found in Table 4.

Here, notice that panel-3 properties no longer produce the best results, but performs slightly worse than both other materials. Additionally, the relative predictions of the different models can give a bit more insight into the other models. Since panel-3 properties cause underprediction of shear, and panel-4 properties cause overprediction of shear, these two models were combined to infer a shear modulus that falls between them. Using weighted average of these two produces an average error of only 29% in the differential cover and 12% in the hemisphere, thus proving that the true shear modulus does, in fact, lie between those measured. As discussed in Section 2, the material variations have been hypothesized to be due to differences in crystallinity due to the rate of cooling during the forming process.

It should be noted here that the errors measured here are larger than those expected for a predictive tool. These predictions, however, would typically not be used as quantitative predictions, but rather as a qualitative indicator of potential problem areas in a given manufacturing process. For this reason it is possible to gain valuable insights even from the generic materials, but any improvement on this can greatly benefit the model’s usefulness. There are a number of possible ways in which this disagreement could arise, which are potentially valuable topics of further research. The first possible source of error is the sliding friction between individual layers, as well as between the blank and tool. Since this was beyond the scope of this research, an approximate friction value was used (as discussed in Section 2). It may be that a better contact model could produce a more accurate simulation. The second possible source of error is in the isothermal nature of the simulations [53]. It is possible, as discussed regarding the differential-cover’s measurements on ‘point-T’, that the cooling as the part interacts with the mold has a significant effect on the shearing behavior. Third, the discrepancies in the shear modulus between different sample panels is likely another source of error. Each of these sources could be investigated, along with possible simple methods of characterization, to further improve the models. Finally, it is possible that some error arose from the limited sample size (two samples for each case-study) of the DIC experiments.

## 4. Structural Analysis of the As-Formed Part Model

In order to conduct structural simulations of the “as-formed” part, we integrated the results of the forming simulation software (PAM-Form) with a structural finite element analysis software (Abaqus) [54]. This is important, since variations in fiber-orientation, fiber-density, and part-thickness influence the strength of any structural member. Properly including this information, which is readily available from forming simulation results, in the structural analysis is expected to make the model more accurate in comparison to the “idealized” part. In order to translate these details between the forming and structural simulation softwares’ formats, a script was developed.

The first step in transferring results of the forming simulation into a structural analysis model involves translating the mesh information from the format used by PAM-Form to that used by the Abaqus software. This begins by gathering the data from the PAM-Form database. One of the main challenges in transferring the results of the forming simulation to a finite element analysis is that PAM-Form simulates a multi-layered blank as many shell-meshes (in order to include sliding contact between layers). While it is possible to model a laminate this way in Abaqus, it requires tie constraints to establish perfect bonding between the layers. This process is made overly complex when the finite element meshes of the individual layers are misaligned, which is inevitable after a forming simulation. Another option would involve tied contact constraints, but this method fails to capture the layer thickness and volume fraction variations caused by matrix migration during the forming process. One solution which can account for this is to first consolidate the layered mesh into a single shell mesh to represent the entire laminate, including fiber orientation and layer-thickness information. This mesh is then used to model the part in Abaqus.

### 4.1. Mesh Conversion Process

**Figure 18:**
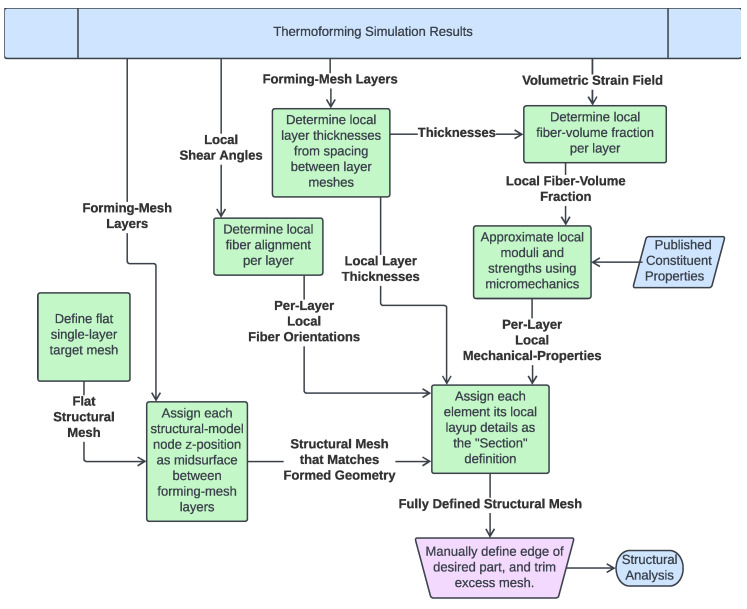
Flowchart of the mesh conversion process.

**Figure 19:**
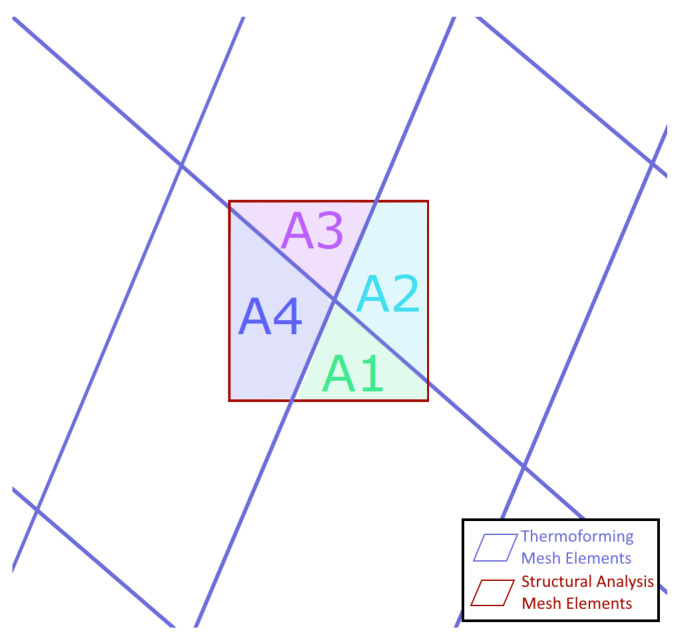
Element area contributions for mesh conversion.

The conversion process was performed using a custom computer program whose function is summarized in Figure 18. To simplifiy the process, a 2D projection is used wherein the z-coordinate of the nodes are neglected when translating the mesh. Ignoring z works nicely for many shapes, but has difficulty in resolving near-vertical faces. Typical stamp formed parts, though, are designed to avoid these kinds of steep inclines, which are difficult to form, making the 2D assumption valid for typical parts. For this reason, we consider these assumptions to be sufficient for this proof of concept, but for completeness, commercial implementations would need to account for steep inclines as well. The remainder of the translation process is summarized by a few critical points. The nodes in the generated single-layer mesh are defined to fall at the midpoint between the multi-layer meshes, thus this single-layer mesh forms the midsurface of the part. Each element in this midsurface mesh is then defined to have composite properties including multiple layers, each of which has its own orientation and thickness. These are 4-noded tetrahedral elements, with their “section” property defined as a laminate whose properties are based on the layers of the mesh from forming simulation. Local thicknesses are determined by taking the spacing between the individual layer-meshes. It is assumed that as long as proper consolidation is maintained, this spacing will be driven primarily by matrix flow and can be captured in the layer thickness parameter. The layer thickness is evaluated separately for each element of the structural mesh. This accounts for thickness and layup variation at every point across the part, including things like dropped plies. Since the meshes typically do not all align, the local orientations are determined using a weighted average of corresponding elements. The weighting factors are determined as the portion of the area of the new element that each contributing element occupies (see Figure 19). The fiber volume fraction is determined using the volumetric strains and thickness change, modified by an "overlap fraction", which takes into account any layer self-overlapping caused by wrinkles. Finally, each layer of the composite is given material properties based on micromechanical approximations using typical properties for polypropylene [55,56] and E-glass [57]. Local elastic properties are approximated using the Mori-Tanaka method [58], while local strengths are defined using a method adapted in [59] from [40,57]. This miromechanics approach was necessary, as forming-induced matrix flow causes the local layer thicknesses and fiber volume fractions to vary across the part, and therefore, any given location possesses different structural properties than the unformed material.

### 4.2. Case Study for Implementing As-Formed Analysis

**Figure 20:**
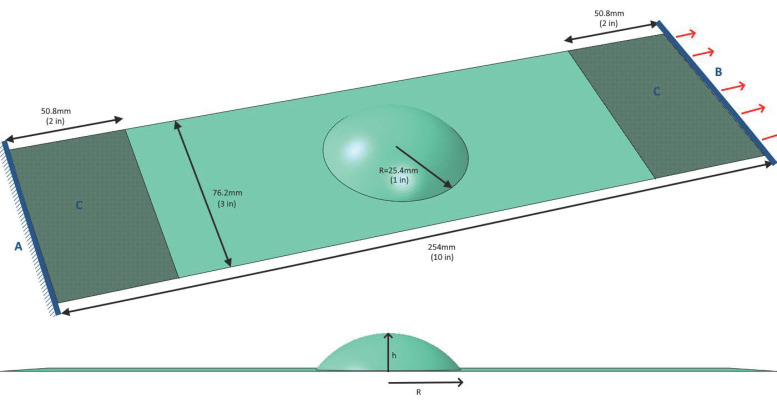
A CAD rendering of the proposed case-study geometry including boundary conditions.

Since the purpose of this study is to demonstrate the value in using as-formed models versus the idealized models (where the fiber alignment and thicknesses are exactly as designed), a case study was required. To this end, a simple geometry was chosen which could be relatively easily formed and tested. This geometry, seen in Figure 20, consists of a flat plate 254 mm × 76.2 mm ($10^{\prime \prime }\times 3^{\prime \prime }$) with an incomplete hemisphere (spherical cap) pressed in the center. This specimen simulates a variety of real-world parts wherein bumps or ridges are added to plates for stiffening. The purpose for this particular form-factor, though, was to produce a part which is not only formable, but also testable, since the plate size is within the limits of a standard tension test frame’s grips.

The hemisphere, which will hereafter be referred to as the “bump”, intersects the plate with a radius of 25.4 mm (1″). The height of the bump was varied as a fraction of this intersection radius between different models, and has been tested for values of h/R= 0.25, 0.5, and 0.75. A model with a full hemispherical bump (h/R = 1) was also attempted, but wrinkled excessively, so as to be unusable. A ${\left[\pm 45^{{}^{\circ}}\right]}_{2}$ layup was used for all models.

#### 4.2.1. Model Specifications

In order to demonstrate the differences between as-formed and idealized modeling, two methods needed to be utilized. First, PAM-Form was used to simulate stamp-forming of the part ( using the weighted-average material properties determined above), the forming results were translated into an Abaqus model, and a structural test was performed. The boundary conditions used were chosen to represent gripping in a tension test machine:

A Displacements and rotations of nodes along one end of the model were fixed.
B Displacements and rotations of nodes along the opposite end, were fixed except for displacement in the direction of the plate’s longitudinal axis. The longitudinal displacement was set to 0.5 mm. This corresponds with a tensile strain of 0.2% in the equivalent flat plate. Note also that this is an elastic quasi-static analysis, so all dynamic effects are considered negligible, and no velocity is defined.
C A region 50.8 mm (2${}^{\prime \prime }$) from either end was fixed to all out-of-plane motion to approximate the restriction caused by a tension tester’s grips.

**Figure 21:**
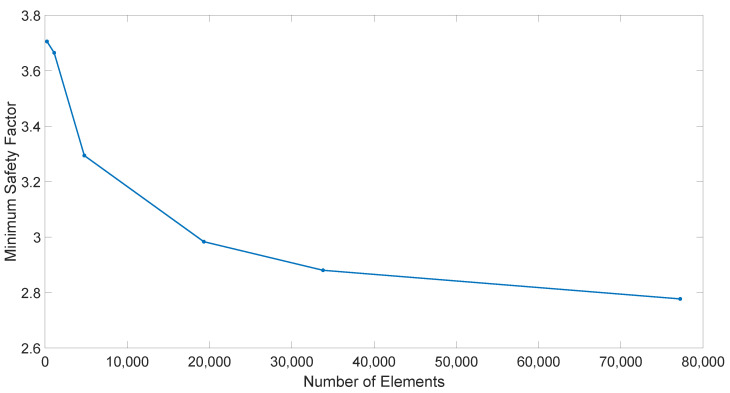
Convergence plot of minimum safety factor (h/R = 0.5).

Next, an equivalent model was generated directly in Abaqus CAE. The fiber directions were assigned to be at $\pm 45^{{}^{\circ}}$ in the global coordinate system, thus presenting an “idealized” part. The part was meshed using 4 node quadrilateral elements (since these are the type generated from the PAM-Form conversion), and the same boundary conditions were used. After a mesh convergence study, a nominal element size of 1 mm was selected for all models. This corresponds to 19,300 elements in Figure 21.

**Figure 22:**
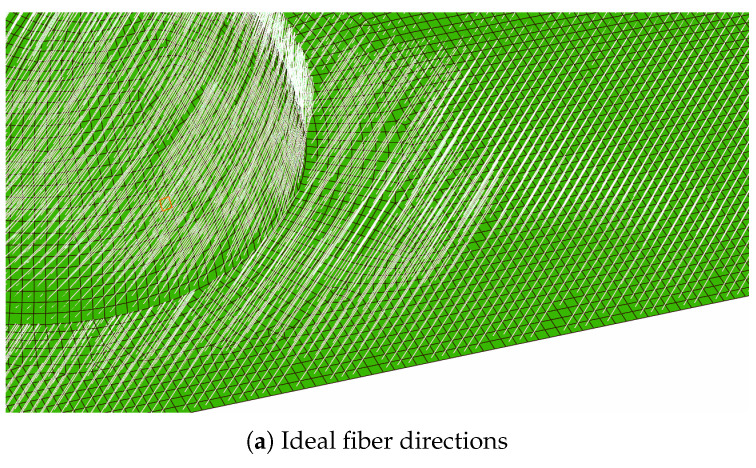

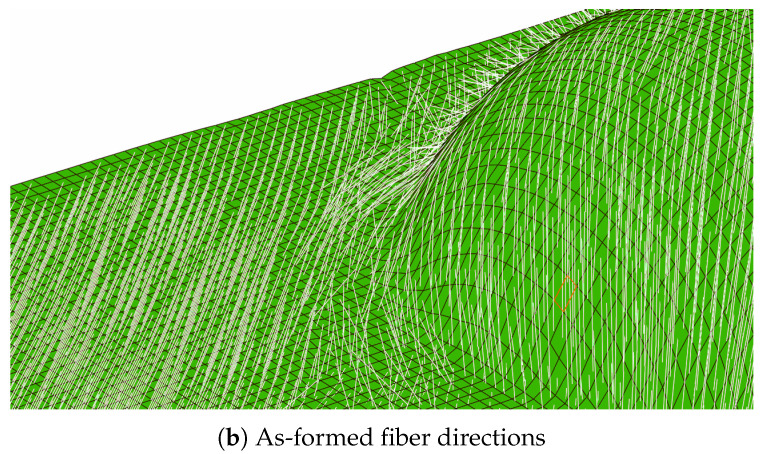
Comparisons of fiber orientation between model types (h/R = 0.75, ${\left[\pm 45^{{}^{\circ}}\right]}_{2}$ layup).

The first thing to look at when inspecting these models is the fiber directions. In the idealized model, the fiber direction is established at the given angle, and is largely unaffected by the bump, whereas for the formed model, the fiber directions near the bump are much different from those further away. This effect becomes more pronounced as the bump height increases, and is particularly vivid for the bump with *h*/*R* = 0.75 as seen in Figure 22, where the fiber directions are rendered as white lines. As can be expected, those models containing a shallow bump demonstrate this effect to a smaller degree, since they require less fiber displacement to form.

#### 4.2.2. Comparison of Stress Concentrations

**Figure 23:**
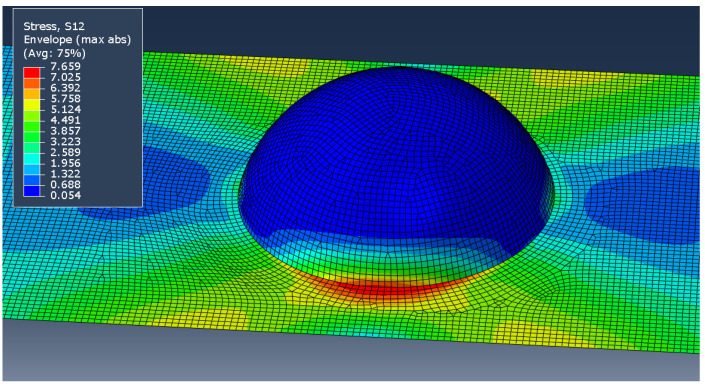
Shear stress (MPa) envelope showing concentration region in an idealized model (*h*/*R* = 0.75, ${\left[\pm 45^{{}^{\circ}}\right]}_{2}$ layup).

The first parameter used to compare the two models was the level of stress concentration near the bump as compared with the stress in an equivalent flat plate (whose stress is given as $\sigma_{0}$). The concentration factor is calculated as $K=\sigma /\sigma_{0}$. Note that the stress field and fiber architecture near the discontinuity in the part are more complicated than can be fully represented by shell elements, so some parts may require a complete 3D analysis for useful predictions. Within the scope of this research, however, it is assumed that the shell assumptions provide a reasonable approximation. Figure 23 shows a representative plot of the in-plane shear stress within the model. While the stresses vary slightly between the layers, and other stress components produce different patterns, they all share the concentration region near the edge of the bump.

**Figure 24:**
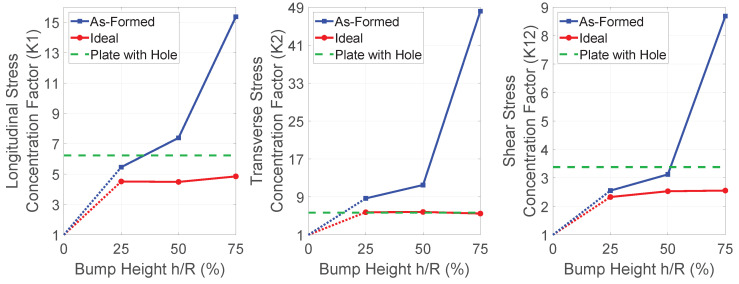
Stress concentration comparisons (${\left[\pm 45^{{}^{\circ}}\right]}_{2}$ layup).

The results of each model were analyzed, and a stress concentration factor generated for each in-plane stress component (longitudinal stress $\sigma_{1}$, transverse stress $\sigma_{2}$, and shear stress $\tau_{12}$). These factors were taken by averaging the stress over several most-stressed elements in each model. Note that the stresses taken were the largest magnitudes seen through the thickness, rather than from an individual layer. The end results are shown in Figure 24.

Looking at these plots, a clear pattern emerges. For the bump with *h*/*R* = 0.25, the ideal and formed part share a similar concentration factor, but as the bump-height increases, the stress concentration factor increases for the formed part at a much larger rate than does the ideal part. This seems to align with intuition, since the smaller bump requires less fiber migration, and therefore the formed and ideal parts are quite similar. Taller bumps require more fiber migration, differentiating the two further.

For comparison, the equivalent model where a hole replaces the bump was also simulated in the same manner, and results are plotted as a horizontal green line in Figure 24. The ideal part generally hovers near or below this value, while the formed-part generally becomes much higher for the larger bumps. This is likely due to fiber migration decreasing the efficiency with which the part can carry load.

In order to determine the effect of layup on the model behavior, the process was repeated using a ${\left[0^{{}^{\circ}}/90^{{}^{\circ}}\right]}_{2}$ layup. These models were generated identically to those discussed above, except the layup.

**Figure 25:**
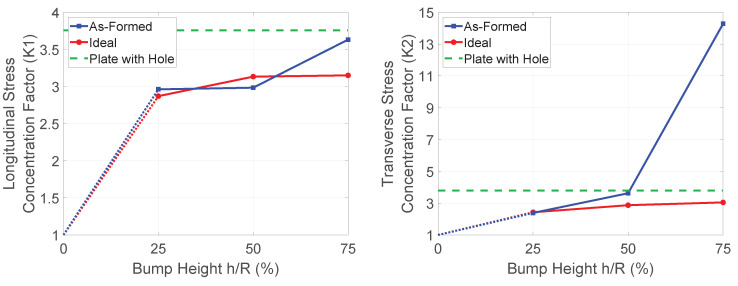
Stress concentration comparisons (${\left[0^{{}^{\circ}}/90^{{}^{\circ}}\right]}_{2}$ layup).

In the ${\left[0^{{}^{\circ}}/90^{{}^{\circ}}\right]}_{2}$ case, there is less difference between models. As seen in Figure 25, the stress concentration factors remain similar for most cases. Note that for a flat plate with ${\left[0^{{}^{\circ}}/90^{{}^{\circ}}\right]}_{2}$ layup, the shear stresses will be negligible. This, then, would create meaningless values of shear stress concentration, so these have been omitted. This indicates that the fiber deviation is a significant factor in determining these values, since the values diverge significantly in those models having significant fiber migration, whereas they remain similar for these models, which have less fiber migration. The only exception to the similarity of concentrations is in the transverse stress in the model with the largest bump, which we attribute to the wrinkles that appeared in the forming model.

#### 4.2.3. Tsai-Wu First Ply Failure

Next, first-ply failure in the part was assessed. To do this, the Abaqus intrinsic Tsai–Wu failure model was implemented. Tsai–Wu uses a quadratic formulation to calculate a failure index within the part [40]. Based on this model, the safety factor for proportional increase of stresses ($S_{f}$) is given as the reciprocal of the failure index. Note that the strength values used here were those determined by micromechanics during the model conversion, and these vary across the part.

**Figure 26:**
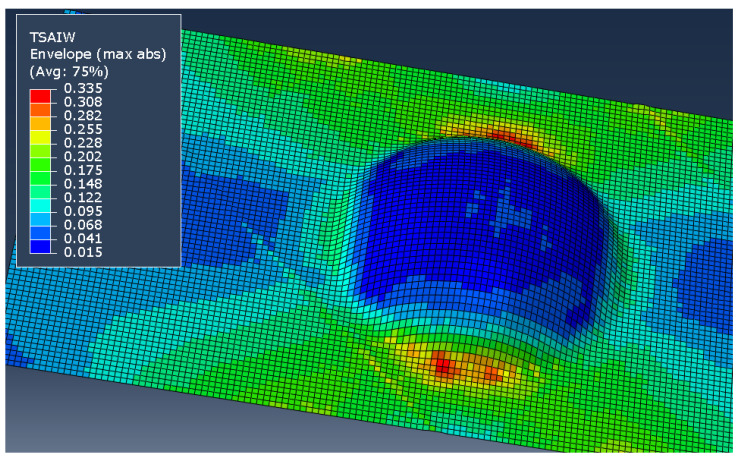
Failure index envelope for the as-formed model (*h*/*R* = 0.5, ${\left[\pm 45^{{}^{\circ}}\right]}_{2}$ layup).

Since the failure index (and thus safety factor) is determined by the relationship between all of the stresses within any given element, it is expected for these metrics to follow similar patterns as the underlying stresses. This pattern can be seen in Figure 26. Based on this, the minimum safety factor has been used to compare the strength to first failure. The data is seen in Table 5, which shows that the as-formed part weakens with larger bumps, whereas the idealized part retains significant strength in all configurations.

Lastly, Table 6 shows the safety factors for the models using a ${\left[0^{{}^{\circ}}/90^{{}^{\circ}}\right]}_{2}$ layup. It is important to note that, since these were modeled in displacement control, so there are different levels of stress depending on the layup. This means that comparing the safety factors between these two layups does not correlate to comparing strengths. It does, however, illustrate the power of this method in allowing the strength of a given part to be predicted.

It is worth noting that the wrinkled model is the weakest by far, having already failed at the applied displacement. This failure is seen to occur within the wrinkled region, as well as happening within a $0^{{}^{\circ}}$ layer (L3) unlike all of the other models which fail in a $90^{{}^{\circ}}$ layer (L2 or L4). Again, this highlights the weakening effect of the wrinkles within the part.

### 4.3. Discussion

These results demonstrate that there is value in using the as-formed geometry in structural analyses. This type of analysis is expected to be very situation dependent, making it crucial for accurate prediction in some processes, and unnecessary for others, with the determining factor being the amount of fiber reorientation. The value of using as-formed fiber geometry for structural analysis is clear [27,28,29], and has recently even been implemented commercially [60,61]. Due to the usefulness of this technique, it will likely become universally utilized in designing complex parts for thermoforming.

## 5. Conclusions and Recommendations

An improved simulation-based thermoforming design process based on the integration of material characterization and as-formed structural analysis has been proposed.

A novel suite of material characterization methods, which relies on DMA techniques, for thermoforming simulations has been implemented and shown capable for measuring several of the required properties. The material model generated by this method has been tested, and after interpolating the shear data, it performed quite well in comparison to the formed parts. This suite, then, can provide an alternative means of material characterization with limited equipment.

The simulation of thermoformed parts using these measured properties in commercial simulation software has been shown to predict critical regions for wrinkling, tearing, and ply separation, and also to predict the shear deformation with reasonable accuracy. This is encouraging in terms of the usability of these methods for industrial design.

A tool for using thermoforming simulation results to inform more accurate structural models has been tested on a simple case study, and produced results that clearly differ from those of ideal models. This provides evidence that this type of analysis may be necessary in some cases. This tool, then, has demonstrated value, and should be investigated for further development and validation as an open source alternative to commercially available solutions.

The tendency of thermoplastic composites to wrinkle has made simulation a must for optimized manufacturing, but the material models required are complex, and time consuming to create. The methods described here have the potential to enhance thermoplastic composites manufacturing by simplifying and expediting the process. In addition, the usefulness of these models is being extended into the realm of bettering predictions of the part’s actual performance, thus improving the design process as well.

This work has demonstrated the value of integrating simple characterization methods with thermoforming simulation toward as-formed structural analysis, but there remain several avenues of research that are beyond the scope of this work, but which would be critical in improving the method toward industrial use. The following recommendations are proposed: Firstly, the discrepancy on shear stiffness between different processing batches warrants further investigation with consideration of the thermal history. Additionally, a friction model which incorporates thermally-varying viscous flow behavior would enhance the accuracy of the simulations. This model would ideally use material properties derived from simple rheometric viscosity experiments. Finally, while isothermal analyses like ours are commonly used, and produce reasonable results [44,53], it is clear that further accuracy could be achieved by implementing a thermal analysis in order to capture the effects of contact-induced local cooling. While analysis software packages often have these thermo-mechanical capabilities, material properties (like thermal conductivity, and heat capacity) are not readily available. A collection of simple methods for measuring these properties would be invaluable for including thermal behavior into the analyses.

## Figures and Tables

**Table 1:** Summary of various material characterization experiments.

Experiment	Target Properties	Specimen Dimensions	Swept Temperatures	Repeats
Dynamic Torsion (Prismatic)	Shear Modulus	10 × 4 × 35 mm	80–200 ${}^{{}^{\circ}}$C	9
Dynamic Torsion (Dogbone)	Shear Modulus	4 × 4 × 35 mm	80–200 ${}^{{}^{\circ}}$C	3
Dynamic Tension (Fiber)	Longitudinal Modulus	8 × 0.3 × 30 mm	40–200 ${}^{{}^{\circ}}$C	8
Dynamic Tension (Transverse)	Transverse Modulus	8 × 0.3 × 30 mm	40–200 ${}^{{}^{\circ}}$C	3
Dynamic Bending (Fiber)	Longitudinal Bending Modulus	15 × 0.3 × 50 mm	40–200 ${}^{{}^{\circ}}$C	8

**Table 2:** Isothermal forming properties for various material models at 180 ${}^{{}^{\circ}}$C.

		Measured Material (Panel-3)	Measured Material (Panel-4)	Measured Material (Panel-5)
$G_{12}$	In-plane shear modulus	21.14 MPa	0.922 MPa	45.19 MPa
$E_{1}^{t}$	Fiber-direction tensile modulus	2.288 GPa		
$E_{1}^{b}$	Fiber-bending modulus	2.288 GPa		
$E_{2}^{t}$	Transverse tensile modulus	4.13 MPa		
$E_{2}^{b}$	Transverse-bending modulus	4.13 MPa		
ρ	Density	1.41 g/cm${}^{3}$		
$V_{fo}$	Initial Fiber Volume Fraction	0.36		

**Table 3:** Shear angle at critical locations in a differential cover.

a Top Surface				
**Region**	**Measured Angle (Experiments)**	**Predicted Angle (Generic Material)**	**Predicted Angle (Measured Material) (Panel 3)**	**Predicted Angle (Measured Material) (Panel 5)**
T	$7.64^{{}^{\circ}}$	$12.0^{{}^{\circ}}$	$15.6^{{}^{\circ}}$	$8.83^{{}^{\circ}}$
FR	$21.2^{{}^{\circ}}$	$29.0^{{}^{\circ}}$	$13.2^{{}^{\circ}}$	$8.02^{{}^{\circ}}$
FL	$21.5^{{}^{\circ}}$	$25.9^{{}^{\circ}}$	$10.9^{{}^{\circ}}$	$8.42^{{}^{\circ}}$
RR	$18.0^{{}^{\circ}}$	$36.1^{{}^{\circ}}$	$12.2^{{}^{\circ}}$	$10.5^{{}^{\circ}}$
RL	$10.8^{{}^{\circ}}$	$32.9^{{}^{\circ}}$	$17.3^{{}^{\circ}}$	$11.0^{{}^{\circ}}$
**b** **Bottom Surface**				
**Region**	**Measured Angle (Experiments)**	**Predicted Angle (Generic Material)**	**Predicted Angle (Measured Material) (Panel 3)**	**Predicted Angle (Measured Material) (Panel 5)**
Region				
T	$6.89^{{}^{\circ}}$	$13.5^{{}^{\circ}}$	$9.72^{{}^{\circ}}$	$7.77^{{}^{\circ}}$
FR	$24.0^{{}^{\circ}}$	$34.1^{{}^{\circ}}$	$11.6^{{}^{\circ}}$	$8.72^{{}^{\circ}}$
FL	$26.0^{{}^{\circ}}$	$23.2^{{}^{\circ}}$	$13.6^{{}^{\circ}}$	$9.00^{{}^{\circ}}$
RR	$19.7^{{}^{\circ}}$	$34.0^{{}^{\circ}}$	$13.5^{{}^{\circ}}$	$11.7^{{}^{\circ}}$
RL	$28.0^{{}^{\circ}}$	$37.8^{{}^{\circ}}$	$11.8^{{}^{\circ}}$	$11.8^{{}^{\circ}}$

**Table 4:** Shear angle at critical locations in a hemisphere.

	Measured Angle (Experiments)	Predicted Angle (Generic Material)	Predicted Angle (Measured Material) (Panel 3)	Predicted Angle (Measured Material) (Panel 4)
Angle	$32.8^{{}^{\circ}}$	$44.6^{{}^{\circ}}$	$19.0^{{}^{\circ}}$	$44.7^{{}^{\circ}}$

**Table 5:** Factors of safety for various models with a ${\left[\pm 45^{{}^{\circ}}\right]}_{2}$ layup.

	Idealized Model		As-Formed Model	
***h*/*R***	$S_{f}^{\min}$	**Ply**	$S_{f}^{\min}$	**Ply**
0.25	4.01	1	3.61	1
0.50	3.62	1	2.98	1
0.75	3.51	1	1.02	2

**Table 6:** Factors of safety for various models with a ${\left[0^{{}^{\circ}}/90^{{}^{\circ}}\right]}_{2}$ layup.

	Idealized Model		As-Formed Model	
***h*/*R***	$S_{f}^{\min}$	**Ply**	$S_{f}^{\min}$	**Ply**
0.25	2.55	2	2.62	2
0.50	2.12	2	1.82	4
0.75	1.96	2	0.65	3

## Data Availability

Not applicable.
